# Cardiovascular Protection with Sulfasalazine During Doxorubicin Challenge: Evidence from Oxidative and Histologic Endpoints

**DOI:** 10.3390/biom16081085

**Published:** 2026-07-24

**Authors:** Onural Ozhan, Enes Kaya, Mehmet Hakan Tasolar, Mehmet Ertugrul Balkar, Azibe Yildiz, Feyzi Dogru, Zeynep Ulutas, Zeynep Kucukakcali, Hakan Parlakpinar

**Affiliations:** 1 Department of Medical Pharmacology, Faculty of Medicine, Inonu University, 44280 Malatya, Türkiye; mertugrul.balkar@inonu.edu.tr (M.E.B.); hakan.parlakpinar@inonu.edu.tr (H.P.); 2 Faculty of Medicine, Inonu University, 44280 Malatya, Türkiye; eneskaya.efk@gmail.com; 3 Department of Cardiology, Faculty of Medicine, Inonu University, 44280 Malatya, Türkiye; hakantasolar@inonu.edu.tr (M.H.T.); zeynep.ulutas@inonu.edu.tr (Z.U.); 4 Department of Histology and Embryology, Faculty of Medicine, Inonu University, 44280 Malatya, Türkiye; azibe.yildiz@inonu.edu.tr; 5 Department of Physiology, Faculty of Medicine, Malatya Turgut Ozal University, 44210 Malatya, Türkiye; feyzi.dogru@ozal.edu.tr; 6 Department of Biostatistics and Medical Informatics, Faculty of Medicine, Inonu University, 44280 Malatya, Türkiye; zeynep.tunc@inonu.edu.tr

**Keywords:** doxorubicin, sulfasalazine, myocardial injury, oxidative stress, aorta, rat

## Abstract

Anthracycline cardiotoxicity involves early oxidative–inflammatory injury to the myocardium and vasculature. Repurposing anti-inflammatory agents may offer pragmatic cardioprotection. The purpose of this study is to evaluate whether sulfasalazine (SSZ) mitigates doxorubicin (DOX)-induced myocardial and aortic injury in rats. Twenty-eight male Wistar albino rats were randomized to: Control (vehicle, *n* = 8), DOX (20 mg/kg i.p., single dose; *n* = 10), and SSZ + DOX (SSZ 300 mg/kg i.p. once daily for 3 days, then DOX 20 mg/kg i.p.; *n* = 10). At 24 h post-DOX, ECG and invasive blood pressure (BP) were recorded. The heart and thoracic aorta were harvested for histopathology and oxidative stress assays (MDA, SOD, GSH, CAT; composite indices where applicable). Compared with SSZ + DOX, the DOX group exhibited higher BP and greater arrhythmic burden. In the aorta, DOX elevated MDA and reduced SOD, GSH, and CAT versus the control, whereas SSZ + DOX shifted these toward control values. In the myocardium, DOX decreased SOD and increased oxidative index; SSZ + DOX attenuated histological injury (edema, hemorrhage, cardiomyocyte degeneration) and restored aortic intima–media thickness toward control. Heart rate was lower in SSZ + DOX than other groups. Short-course SSZ pretreatment alleviated early DOX-induced oxidative stress and structural damage in myocardial and aortic tissues, with concurrent improvement in hemodynamic and ECG profiles. These data support further dose–timing optimization and longer-term functional studies to define SSZ’s translational potential as an adjunct cardioprotective strategy during anthracycline exposure.

## 1. Introduction

Cancer therapy-related cardiac dysfunction (CTRCD) is increasingly recognized as a major complication of modern cancer treatment, encompassing structural and functional cardiac abnormalities that may develop during or after exposure to anticancer therapies. Anthracyclines, particularly doxorubicin (DOX), are among the most common causes of CTRCD, underscoring the ongoing need for effective cardioprotective strategies [1]. DOX remain indispensable in the treatment of a broad spectrum of malignancies, yet their clinical utility is limited by acute and chronic cardiotoxicity that can present as myocarditis, arrhythmias, and progressive cardiomyopathy. Contemporary cardiology guidance underscores the diagnostic and prognostic relevance of inflammatory myocardial injury in this setting and highlights the unmet need for mechanism-based cardioprotection during cancer therapy [2,3,4,5]. The pathogenesis of DOX-induced myocardial damage is multifactorial, involving topoisomerase II-β-mediated DNA injury, mitochondrial dysfunction with excessive reactive oxygen species (ROS) generation, iron-dependent oxidative damage, calcium dysregulation, and pro-inflammatory signaling. Mitochondria are central hubs in this injury cascade; altered mitochondrial dynamics, impaired oxidative phosphorylation, and metabolic inflexibility amplify oxidative stress and cardiomyocyte death [6,7,8]. On the other hand, rats are generally preferred because the immune, physiological, and cardiac responses they exhibit in the myocardial injury model closely reflect the progressive organ damage observed in humans with DOX-induced cardiotoxicity, providing a strong translational advantage.

Oxidative stress (OS) is tightly linked to DOX cardiotoxicity: increased lipid peroxidation (e.g., malondialdehyde, MDA) coincides with depletion or dysfunction of endogenous antioxidant defenses, including superoxide dismutase (SOD), catalase (CAT), and glutathione (GSH). Modulating this redox imbalance, by limiting ROS burden and/or reinforcing antioxidant capacity, remains a rational therapeutic strategy to mitigate DOX-related myocardial injury [9].

Sulfasalazine (SSZ), a long-standing anti-inflammatory agent used in inflammatory bowel disease and rheumatoid arthritis, exerts pleiotropic actions that are mechanistically relevant to DOX cardiotoxicity. SSZ is a potent, relatively specific inhibitor of nuclear factor kappa B (NF-κB) activation—acting in part through inhibition of IκB kinase (IKK) activity—thereby dampening downstream inflammatory gene expression [10,11]. In addition, SSZ inhibits the system x_c_^−^ cystine/glutamate antiporter (xCT), limiting cystine uptake for GSH synthesis and modulating redox signaling in inflamed or stressed tissues [12,13]. Together, these properties position SSZ as a candidate to attenuate both inflammatory and oxidative components of anthracycline cardiotoxicity.

SSZ has been shown to directly scavenge a range of ROS and reactive nitrogen species (RNS) in validated in vitro assays, supporting its antioxidant properties [14]. In animal models of colitis and other inflammatory conditions, SSZ administration significantly reduced markers of OS and restored antioxidant enzyme activities in tissues. Studies comparing SSZ to other agents consistently found that SSZ alone improved antioxidant status and reduced oxidative tissue damage, though some combinations produced even greater effects [15,16,17].

Laboratory and ex vivo studies suggest SSZ can reduce endothelial dysfunction, promote blood vessel dilation, and enhance endothelial cell migration and proliferation. These effects were observed in human placental and vascular tissues, indicating possible vasoprotective properties, though these findings have not been confirmed in large clinical trials for cardiovascular disease [18,19].

Despite its mechanistic appeal, the potential of SSZ to counteract DOX-induced myocardial injury has not been systematically investigated in vivo. We therefore tested the hypothesis that SSZ pretreatment would mitigate DOX-evoked OS, electrical and hemodynamic disturbances, and histopathological myocardial injury in rats using a well-established experimental model.

## 2. Materials and Methods

### 2.1. Study Design

Twenty-eight male Wistar albino rats, weighing 294–467 g and aged 17 weeks, were procured from the Inonu University Laboratory Animals Research Center and put in a temperature (21 ± 2 °C) and humidity (60 ± 5%) controlled environment with a 12:12 h light/dark cycle. The rats were provided ad libitum a standard chow pellet diet with tap water. To minimize potential confounding factors, standard laboratory conditions and a consistent experimental layout were rigorously maintained. The animals were socially grouped, with a maximum of four rats per cage, and housed under the same environmental conditions with a uniform cage layout between groups to prevent positional bias. To minimize potential confounding effects related to the time of day and treatment order, animals from all experimental groups were processed in an alternating sequence throughout each experimental session. Thus, no single group was treated or assessed as a complete batch before the others. All treatments, physiological measurements, tissue collection, and sample processing were performed under identical experimental conditions using standardized protocols by the same investigator.

All of the experiments in this study were approved by the Committee on Animal Research (reference no. 2023/5-6) at Inonu University in Malatya, Türkiye. All methods were carried out in accordance with relevant guidelines and regulations. The National Institutes of Health’s Guide for the Care and Use of Laboratory Animals and Animals in Research: Reporting In Vivo Experiments (ARRIVE) criteria were followed in all experiments and procedures conducted for this work [20,21].

Predefined inclusion or exclusion criteria were not applied to the animals used in this study. A total of 28 animals participated in the experiment. There were no exclusions for data analysis at the end of the experiment. Additionally, a partial double-blind method was used in this study. The responsible author was the person in charge of assigning the animals to groups, conducting the experiment, and measuring the outcomes, and was aware of the group assignments. However, to ensure objectivity and eliminate potential bias during data analysis, the researcher conducting the statistical analysis was kept unaware of the group assignments.

Twenty-eight rats were divided into three groups. The rats were assigned to their respective cages and experimental groups using a simple randomization procedure based on computer-generated random numbers. Each rat was assigned a unique identification number before randomization, and group allocation was completed before treatment administration.

Control group (*n* = 8): Saline was administered intraperitoneally (i.p.) at a volume of 10 mL/kg as the vehicle solution. DOX group (*n* = 10): A single dose of 20 mg/kg DOX i.p. was administered. SSZ + DOX group: Following administration of a single daily dose of 300 mg/kg SSZ i.p. for 3 days, a single dose of 20 mg/kg DOX i.p. was administered. The dose, route, and intervals of administration of DOX (Adrimisin^®^ Lyophilized Powder for Injection, 10 mg Vial, Saba Pharmaceuticals, Istanbul, Türkiye), SSZ (CAS Number: 599-79-1, Sigma-Aldrich, St. Louis, MO, USA) and the vehicle solution were determined with reference to previous studies [22,23]. No animals died or developed severe adverse effects during the experimental period. Following DOX administration, transient red discoloration of the urine was observed in some animals, consistent with the known pharmacological properties of DOX, and no intervention or early euthanasia was required.

### 2.2. Euthanasia and Tissue Collection Procedure

The rats were weighed at the beginning and end of the experiment. To ensure complete loss of consciousness and minimize any potential pain or distress during tissue harvesting, all rats were deeply anesthetized with ethyl carbamate (1.2 g/kg, i.p.) (urethane; Acros Organic, Geel, Belgium) 24 h after the DOX administration. Under anesthesia, the animals were euthanized by surgical exsanguination during tissue collection. The heart and thoracic aorta tissues were collected. Heart weight was measured with a sensitive scale. Heart and aorta tissues were divided into two symmetrical parts for histopathological and biochemical analyses. Heart and aorta tissue reserved for biochemical analysis is stored in a −80 °C deep freezer, while heart and aorta tissue reserved for histopathological analysis is fixed in 10% formaldehyde.

### 2.3. Hemodynamic Parameters

At the end of the experiment systolic, diastolic and mean blood pressure (BP) invasively were measured with a cannula inserted into the carotid artery. Heart rate (HR) and electrocardiography (ECG) were determined. Under general anesthesia, rats were monitored for HR, BP, and ECG using the Biopac MP100 data acquisition system (Biopac Systems Inc., Santa Barbara, CA, USA). ECG signal activity was collected for at least 3 min at a sampling frequency of 500 Hz using disposable electrodes placed on the rat’s thorax. After the recordings were completed, the ECG traces were visually evaluated by two specialists to determine HR and severe ECG abnormalities such as arrhythmia, ST elevation, ST depression, T negativity, and aberrant conduction using the Lambeth Convention diagnostic criteria [24]. In addition, the analysis looked at the high-precision measurements of the duration and fluctuations in PR, QRS, and QT interval variability between the groups.

### 2.4. Biochemical Analysis

#### 2.4.1. Tissue Biochemistry

When the analyses began, the tissues were rinsed by immersing them in a beaker containing Tris-HCl. Their weights were then measured and recorded. Tissues placed in glass tubes were homogenized for one minute using a T 25 B homogenizer (IKA-Werke GmbH & Co. KG, Staufen, Germany) after adding pH 7.4 Tris-HCl buffer. Homogenization was completed by adding a little more buffer solution and homogenizing for another minute. A portion of the resulting homogenate was set aside for analysis. The remaining homogenate was centrifuged at 4000 rpm for 45 min at +4 °C in a refrigerated centrifuge (Andreas Hettich GmbH & Co. KG, Tuttlingen, Germany). The clear supernatant was separated for analysis. The relevant spectrophotometric readings were performed using UV-160A spectrophotometer (Shimadzu Corporation, Kyoto, Japan) and Synergy LX multimode microplate reader (BioTek Instruments, Inc., Winooski, VT, USA) devices.

#### 2.4.2. Malondialdehyde (MDA) Measurement

MDA was measured according to the method of Uchiyama and Mihara [25]. The method is based on the combination of MDA, a lipid peroxidation product, with thiobarbituric acid at 95 °C. After applying the procedures to the standards and homogenate, the n-butanol phase separated in the tubes belonging to the samples was read with a spectrophotometer at 535 and 520 nm wavelengths, and the difference was recorded. The results obtained were expressed in nmol/g tissue.

#### 2.4.3. Glutathione (GSH) Measurement

The GSH concentration in the homogenate was measured spectrophotometrically using the Ellman method [26]. Each homogenate sample was mixed with 10 mM 5,5-dithiobis (2-nitrobenzoic acid) in 100 mM potassium phosphate buffer (pH 7.5) and 17.5 M ethylenediaminetetraacetic acid (EDTA). The reaction was initiated by adding 0.5 units of glutathione reductase and 0.4 mM nicotinamide adenine dinucleotide phosphate (NADPH). After 5 min, the absorbance of the samples was measured at 410 nm, and the GSH concentration was calculated according to a standard curve. The results were expressed as μmol/g tissue.

#### 2.4.4. Superoxide Dismutase (SOD) Activity Measurement

SOD activity was determined using the method of Sun et al. [27]. The SOD activity test is based on the principle of inhibition of Nitroblue Tetrazolium Chloride (NBT) reduction. SOD activity is inversely proportional to the absorbance value of formazan at 560 nm. Results were calculated in U/g protein.

#### 2.4.5. Protein Quantification

Protein quantity analysis was performed using the modified Lowry method to calculate the data for the other markers studied [28]. Folin reagent was added to the alkaline copper-protein solution containing the samples and vortexed. This application ensured that the reduction reaction occurred before the folin reagent decomposed. The standards and samples were read at a wavelength of 750 nm in a spectrophotometer. The results were calculated according to the standard graph obtained and expressed in μg/mL.

#### 2.4.6. Measurement of Catalase (CAT) Activity

CAT activity was determined according to Aebi’s method [29]. The test principle is based on determining the rate constant (k, 1/s) or the hydrogen peroxide (H_2_O_2_) decomposition rate at a wavelength of 240 nm. Decomposition begins with the addition of supernatant to the H_2_O_2_ solution, and the change is monitored in a spectrophotometer. This change was monitored for a specific period, and the activity and rate constant were calculated based on the absorbance change. Results were reported as K/g protein.

#### 2.4.7. Glutathione Peroxidase (GPx) Activity Measurement

GPx activity was measured using the Paglia and Valentine method [30]. An enzymatic reaction was initiated by adding H_2_O_2_ to tubes incubated with supernatant added to a tube containing NADPH, reduced glutathione, sodium azide, and glutathione reductase. This reaction was monitored at 340 nm wavelength using a spectrophotometer for a specific period of time. The activity calculated from the observed absorbance change during this process was given as U/mg protein.

#### 2.4.8. Calculation of the Oxidative Stress Index (OSI) Using Total Antioxidant Status (TAS) and Total Oxidant Status (TOS) Measurements

TAS and TOS measurements were performed using specially developed Rel Assay Diagnostics (MEGA TIP San. Tic. Ltd. Sti., Gaziantep, Türkiye) brand test kits, following the manufacturer’s instructions, and calculated as specified in the manual.

The TAS measurement was determined in the supernatant using the Erel method [31]. The method is based on the oxidation of the 2,2′-azinobis-3-ethylbenzothiazoline-6-sulfonic acid (ABTS) molecule to the ABTS^+^ molecule in the presence of H_2_O_2_. In this measurement method, the ABTS radical is used. The ABTS radical loses its blue and green color depending on the antioxidant amount and antioxidant capacity. This color change is measured at a wavelength of 660 nm for evaluation. There is an inverse relationship between the color change and the antioxidant amount in the sample. The reaction rate is adjusted using Trolox, a well-known method. The unit is mmol Trolox equivalent/L. TOS was measured colorimetrically according to the Erel method using the supernatant obtained [32]. The method is based on the oxidation of the ferrous ion-o-dianisidine complex of oxidants to ferric ion. The presence of glycerol molecule in the reaction medium is necessary for oxidation to occur. In acidic medium, ferric ions form a colored complex with xylenol orange chromogen. At a wavelength of 530 nm, the intensity of this complex is measured with a spectrophotometer. The unit is μmol H_2_O_2_ equivalent/L. The OSI was calculated by dividing the TOS by the TAS. The unit is arbitrary unit (AU) [33].

### 2.5. Histopathological Analyses

Heart and aorta tissues obtained at the end of the experiment were fixed in 10% formaldehyde. Sections 4 µm thick were taken from paraffin blocks prepared after tissue tracking procedures. The sections were stained using the hematoxylin–eosin staining method to determine the general morphological structure. Evaluations were performed using a Leica DFC-280 research microscope and the Leica Q Win Image Analysis System (Leica Micros Imaging Solutions Ltd., Cambridge, UK). Heart sections were evaluated for hemorrhage, interstitial edema, and cardiomyocyte degeneration (dense eosinophilic cytoplasm, pyknotic nucleus). Ten randomly selected areas were examined and scored according to the degree of histological changes: 0: no change, 1: mild, 2: moderate, 3: severe change. For the evaluation of the aorta, the thickness of the tunica intima-media was measured in five randomly selected areas from three separate sections taken from each sample.

### 2.6. Statistical Analyses

The power analysis performed calculated that the minimum sample size required for the study is 8 per group, totaling 24. Since a 25% mortality rate could be observed in rats with myocardial injury induced by DOX, the groups to be induced with myocardial injury were determined to be *n* = 10, totaling 28.

SPSS software (IBM Statistics Version 25) was used for the statistical analysis of the obtained data. The normality of quantitative data was assessed using the Kolmogorov–Smirnov test. For indicators that met the normality assumption, between-group differences in quantitative variables were analyzed using one-way ANOVA, and multiple comparisons were performed using Tukey HSD. When the assumption of normality was not met, differences between groups in terms of quantitative variables were assessed using the Kruskal–Wallis H test. Quantitative data in the study were presented as mean ± standard deviation for parametric tests and as median (minimum–maximum) for non-parametric tests. Categorical variables, including ECG findings, were analyzed using Fisher’s exact test. A *p* value < 0.05 was considered statistically significant.

## 3. Results

### 3.1. Body and Heart Weights of Rats

A total of 28 rats were included in the study. The beginning of the experiment, the end of the experiment, and the heart weights of the rats included in the study are summarized in Table 1. There was no significant difference between the pre- and post-experiment groups in terms of weights. Although the overall body weight range of the animals was relatively broad, randomization resulted in balanced baseline body weights across the study groups, and no statistically significant difference was detected before treatment initiation (Table 1, *p* = 0.293).

### 3.2. Hemodynamic Parameters

**Figure 1:**
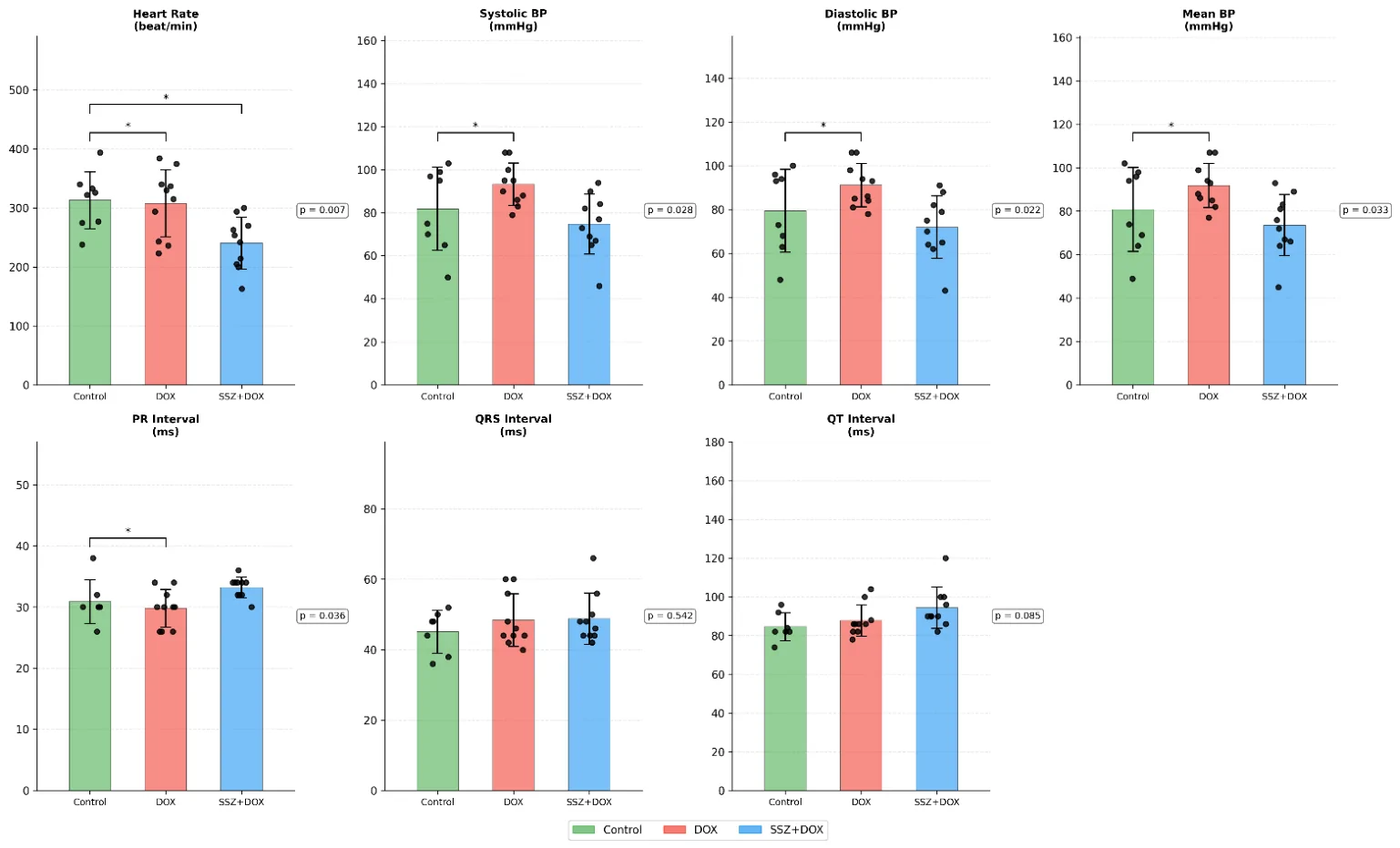
Effects of SSZ pretreatment on DOX-induced changes in HR, systolic BP, diastolic BP, mean BP, PR interval, QRS interval, and QT interval. Data are presented as mean ± SD. Control group, *n* = 8; DOX group, *n* = 10; SSZ + DOX group, *n* = 10. Between-group differences were analyzed using one-way ANOVA followed by Tukey’s HSD post hoc test. Horizontal lines indicate the specific group comparisons. * *p* < 0.05.

The hemodynamic and electrocardiographic data of the study population are presented in Figure 1. In particular, systolic BP, diastolic BP, mean BP, and PR intervals were statistically significantly higher in the DOX group compared to the SSZ + DOX group (*p* < 0.05). In addition, HR was significantly lower in the SSZ + DOX group compared to the other groups (*p* < 0.05).

#### ECG Changes

**Figure 2:**
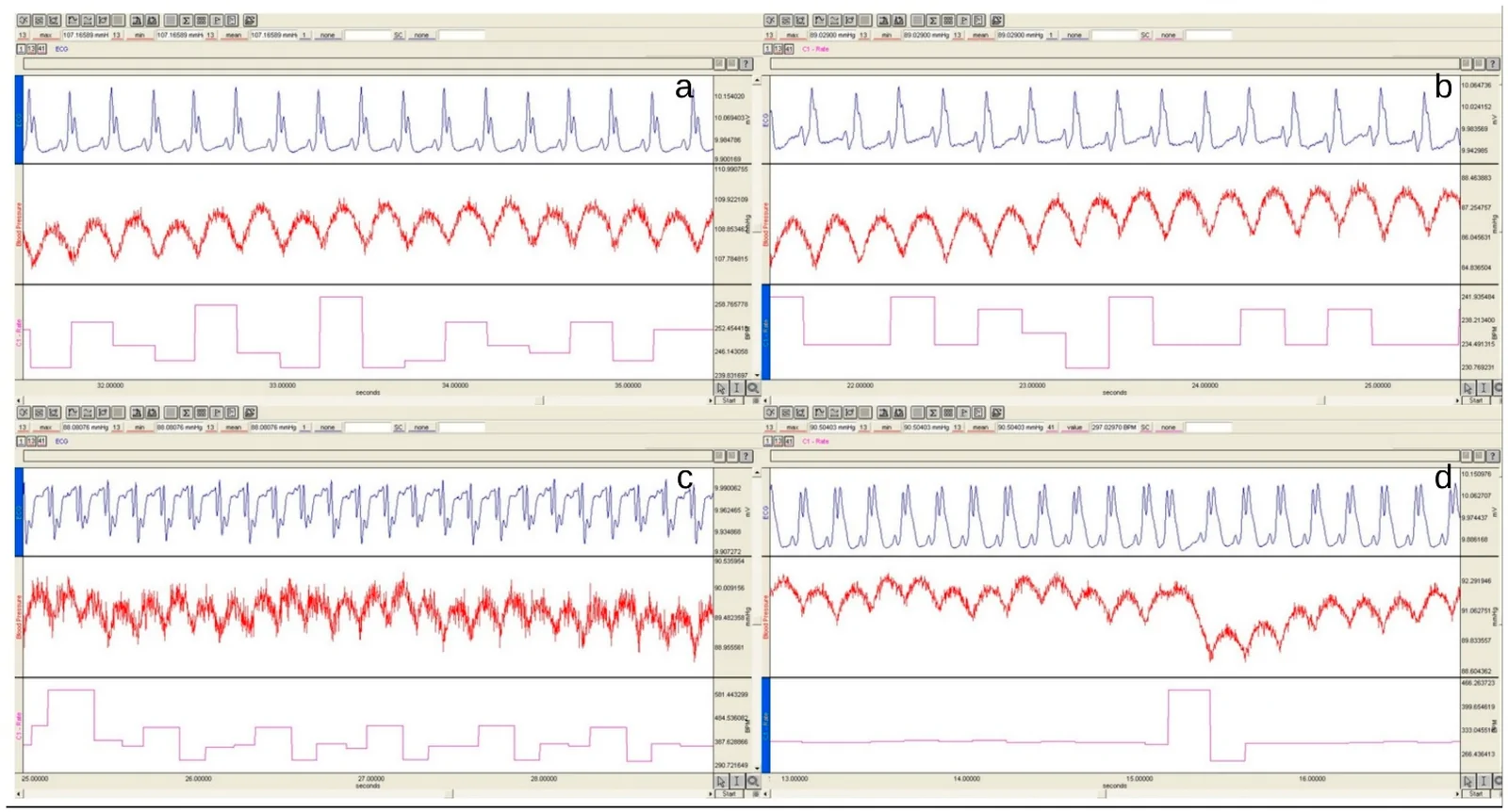
ECG changes of rats. (**a**) Normal sinus rhythm, (**b**) ST-segment elevation detected in a rat from the DOX group, (**c**) ST-segment depression and T-wave inversion detected in a rat from the DOX group, (**d**) an atrial extrasystole detected in a rat from the SSZ + DOX group.

The frequencies of ECG abnormalities among the experimental groups were compared using Fisher’s exact test (*p* > 0.05). The group exhibiting the highest incidence of ECG abnormalities (Figure 2, Table 2) was the DOX group. In this group arrhythmia (sinus tachycardia), T wave negativity and ST segment depression (Figure 2c) was detected in 10% of rats (1 out of 10), ST segment elevation (Figure 2b) was detected in 30% (3 out of 10). In the SSZ + DOX group, arrhythmia (bradycardia, atrial extrasystole) was detected (Figure 2d) in 30%, ST segment elevation and aberrant conduction were observed in 10% (1 out of 10) of rats.

### 3.3. Biochemical Analysis

#### 3.3.1. Heart Tissue Biochemical Analysis

**Figure 3:**
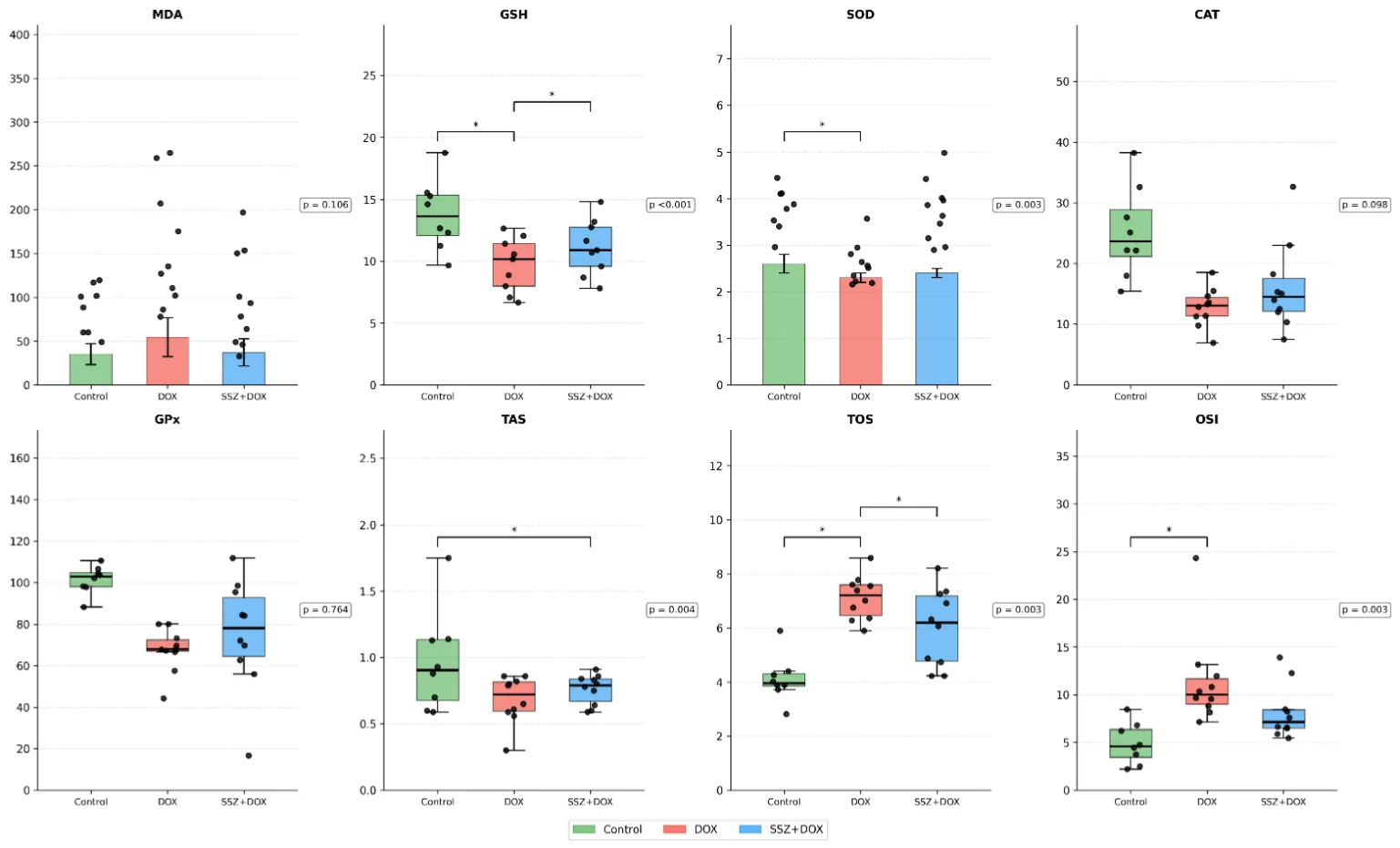
Effects of SSZ pretreatment on OS and antioxidant defense parameters in heart tissue following DOX administration. Data are presented as mean ± SD or median with minimum–maximum values, according to data distribution. Control group, *n* = 8; DOX group, *n* = 10; SSZ + DOX group, *n* = 10. Normally distributed variables were analyzed using one-way ANOVA followed by Tukey’s HSD test, whereas non-normally distributed variables were analyzed using the Kruskal–Wallis test followed by Mann–Whitney U test. Horizontal lines indicate statistically significant comparisons between groups. * *p* < 0.05.

When biochemical analyses obtained from heart tissue were evaluated, the SOD value was lower in the DOX group compared to the control group, while the OSI value was significantly higher than in the control group (*p* < 0.05). The TAS value was statistically significantly lower in the DOX group compared to the SSZ + DOX group (*p* < 0.05). In addition, although this value was also lower in the DOX group compared to the control group, this difference did not reach statistical significance (*p* > 0.05). The GSH value was significantly lower in the DOX and SSZ + DOX groups compared to the control group, while the TOS value was significantly higher in both groups compared to the control group (*p* < 0.05) (Figure 3).

#### 3.3.2. Aorta Tissue Biochemical Analysis

**Figure 4:**
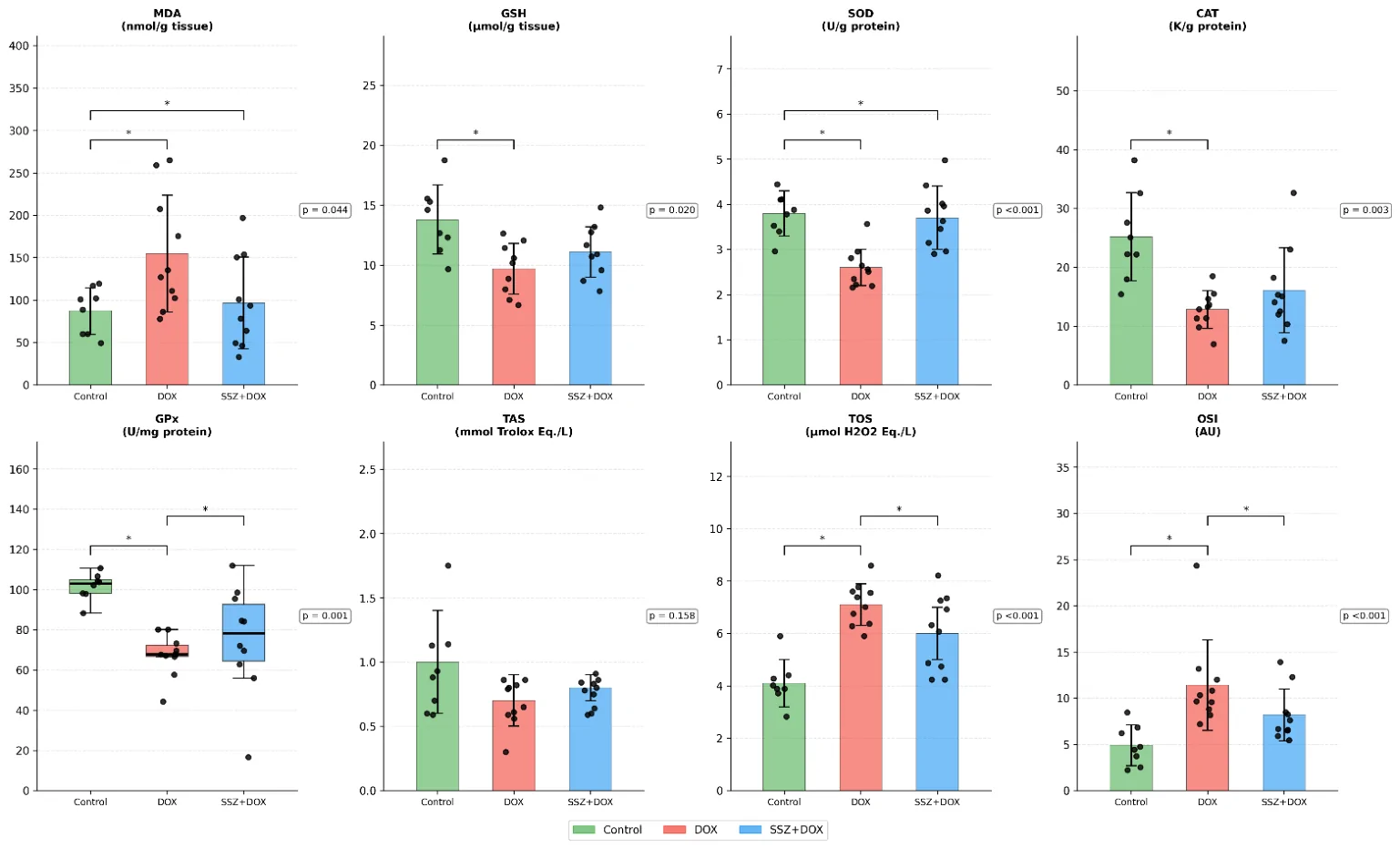
Effects of SSZ pretreatment on OS and antioxidant defense parameters in aortic tissue following DOX administration. Data are presented as mean ± SD or median with minimum–maximum values, according to data distribution. Control group, *n* = 8; DOX group, *n* = 10; SSZ + DOX group, *n* = 10. Normally distributed variables were analyzed using one-way ANOVA followed by Tukey’s HSD test, whereas non-normally distributed variables were analyzed using the Kruskal–Wallis test followed by Mann–Whitney U test. Horizontal lines indicate statistically significant comparisons between groups. * *p* < 0.05.

When analyzing aortic tissue, MDA values were higher in the DOX group compared to other groups, while SOD values were significantly lower in the DOX group compared to other groups (*p* < 0.05). Similarly, GSH and CAT values in aortic tissue were lower in the DOX group compared to the control group (*p* < 0.05). The GPx value was significantly lower in the DOX and SSZ + DOX groups compared to the control group, while the TOS and OSI values were significantly higher in both groups compared to the control group (*p* < 0.05) (Figure 4).

### 3.4. Histopathological Analyses

#### 3.4.1. Myocardium Tissue

**Figure 5:**
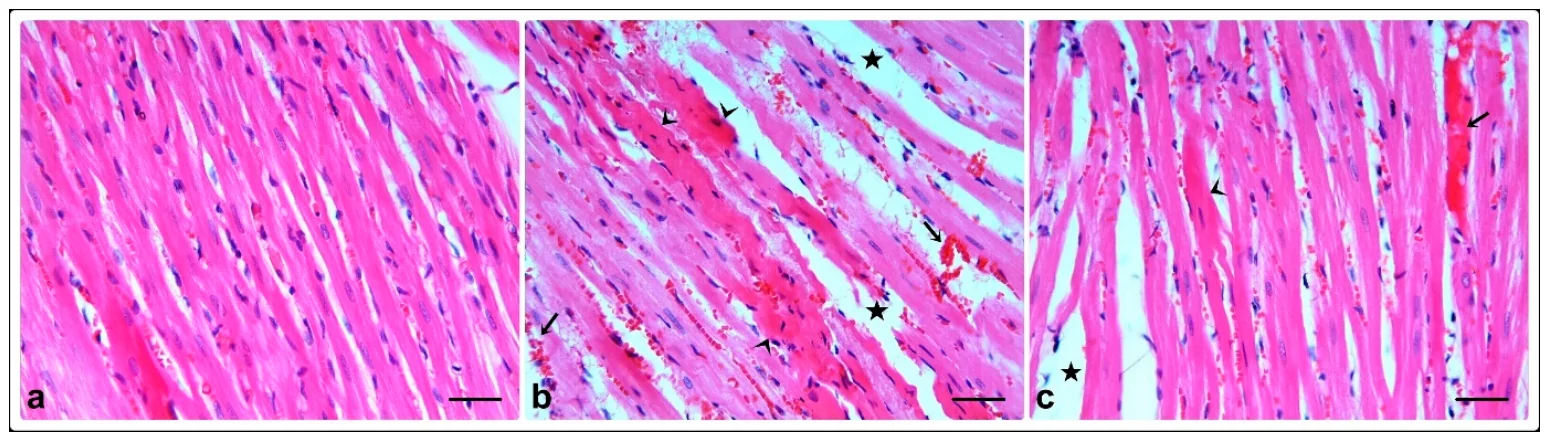
The control group (**a**) shows normal histological appearance except for mild edema and hemorrhage in the myocardial tissue. In the DOX group (**b**), increased interstitial edema (stars), widespread hemorrhage (arrows), and cardiomyocyte degeneration (arrowheads) are observed in the myocardium. In the SSZ + DOX group (**c**), edema (stars), hemorrhage (arrows), and cardiomyocyte degeneration (arrowheads) are markedly reduced compared to the DOX group. H&E staining, ×400 magnification; scale bar = 50 µm.

Except for mild changes in the control group, the myocardium showed a normal histological appearance (Figure 5a). On the other hand, in the DOX group, interstitial edema, hemorrhage, and widespread cardiomyocyte degeneration, particularly in hemorrhagic areas, were noted (Figure 5b). The histopathological changes observed in the DOX group were found to be statistically higher than those in the control group (*p* < 0.001). SSZ administration significantly attenuated the histopathological changes caused by DOX (*p* < 0.001), and the myocardial tissue in the SSZ + DOX group appeared almost similar to that in the control group. (Figure 5c). The results of the histopathological evaluation of the myocardium are presented in Table 3.

#### 3.4.2. Aorta Tissue

**Figure 6:**
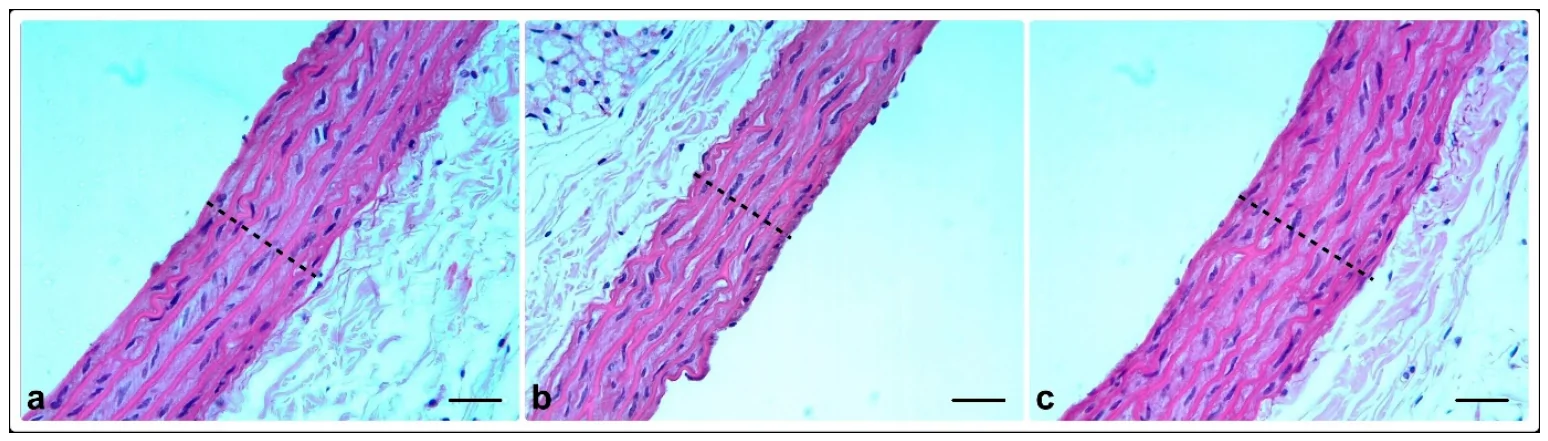
Appearance of the vessel wall in the control group (**a**), DOX group (**b**), and SSZ + DOX group (**c**), respectively. The black line indicates the thickness of the tunica intima-media. H&E staining, ×400 magnification; scale bar = 50 µm.

All groups exhibited normal histological structure of the vessel wall; however, in the DOX group, the thickness of the tunica intima-media was found to be statistically significantly lower than in the control group (*p* = 0.006). In the SSZ + DOX group, the tunica intima-media thickness was found to be statistically higher than in the DOX group (*p* < 0.001, Figure 6). The tunica intima-media thickness of each group is shown in Table 4.

## 4. Discussion

DOX remains one of the most effective chemotherapeutic agents against a wide spectrum of hematologic and solid malignancies. However, its clinical utility is severely limited by dose-dependent cardiotoxicity, which can manifest acutely as myocarditis or chronically as irreversible dilated cardiomyopathy [2,3,4,5]. The pathogenesis of DOX-induced cardiac injury is multifactorial but centers on mitochondrial dysfunction, excessive generation of ROS, iron-mediated oxidative damage, calcium mishandling, and activation of pro-inflammatory signaling cascades, particularly NF-κB [6,7,8]. In this context, therapeutic strategies that simultaneously target OS and inflammation represent a rational approach to cardioprotection. Our study demonstrates for the first time that SSZ, a clinically approved anti-inflammatory drug, significantly attenuates DOX-induced myocardial and vascular injury in a rat model, primarily through restoration of redox homeostasis and preservation of tissue architecture.

In our model, a single high-dose administration of DOX induced significant hemodynamic perturbations, including elevated systolic, diastolic, and mean arterial BP compared to both control and SSZ-pretreated animals. This hypertensive response may reflect DOX-induced sympathetic overactivation, endothelial dysfunction, or vascular hyperreactivity—phenomena previously reported in rodent models of anthracycline toxicity [4]. Notably, SSZ pretreatment (300 mg/kg/day for 3 days prior to DOX) normalized BP parameters, suggesting a vasculoprotective effect. This aligns with prior evidence that SSZ can improve endothelial function in diabetic rats and reduce vascular inflammation via NF-κB inhibition [19]. In a mouse model of preeclampsia, SSZ produced a modest, short-term decrease in diastolic and mean BP, but this effect was not sustained throughout gestation and did not affect systolic BP. The study concluded that SSZ did not provide a lasting reduction in BP in this context [34]. Laboratory experiments using human placental tissues and blood vessels found that SSZ improved endothelial function and promoted vasodilation in pre-constricted arteries, suggesting potential vascular benefits. However, these findings were limited to in vitro and ex vivo settings and did not directly measure systemic BP in humans [18]. No clinical trials or observational studies were identified that directly demonstrate a decrease in BP in humans associated with SSZ use for any indication, including preeclampsia, autoimmune diseases, or cardiovascular conditions [18,34,35]. In addition, the comparatively narrow pulse pressure observed in all three groups may be attributable to the effects of general anesthesia [36,37].

Concurrently, the SSZ + DOX group exhibited a significantly lower HR compared to both the control and DOX-only groups. While bradycardia is not a typical feature of DOX cardiotoxicity, SSZ’s ability to modulate autonomic tone or directly influence sinoatrial node activity cannot be excluded. Current research does not report a decrease in HR as an effect of SSZ use. Large population-based studies in patients with ankylosing spondylitis have shown that SSZ is associated with a reduced risk of major adverse cardiovascular events (MACE), but these studies do not mention HR changes as an outcome or adverse event [38,39]. Furthermore, the ECG changes observed in this study clearly reflect the manifestations of acute DOX-induced cardiotoxicity. The ST-segment changes, T-wave abnormalities, and sinus tachycardia observed in animals treated with DOX indicate the direct damage caused by this agent to myocardial tissue, coronary perfusion disorders, and deviations in ventricular repolarization mechanisms [40]. The ECG patterns observed, consequent to the acute damage induced by the drug through disruption of myocardial membrane integrity, indicate that the model has been successfully established and point to a significant underlying cardiac injury. Of particular interest was the observation that the incidence of arrhythmias, specifically atrial extrasystoles and bradycardic episodes, was most prevalent in the SSZ + DOX group. Although this may appear paradoxical, given that ST-segment abnormalities, T-wave inversions and conduction blocks were not observed with greater frequency in the SSZ group, this finding may reflect the interaction between reduced heart rate and increased vagal tone, rather than myocardial damage leading to arrhythmia. Moreover, the absence of serious ventricular arrhythmias lends further support to the hypothesis that SSZ does not exacerbate electrical instability in this acute setting. A case report documented the development of ventricular tachycardia in a newborn infant due to hyperkalemia associated with SSZ exposure during pregnancy. The arrhythmia resolved with treatment for hyperkalemia, suggesting an indirect link via electrolyte imbalance rather than a direct arrhythmogenic effect of SSZ itself [41].

The prolonged PR interval in the SSZ + DOX group further suggests a mild delay in atrioventricular conduction, possibly mediated through anti-inflammatory effects on nodal tissue or altered calcium handling. While such changes warrant caution in clinical translation, they do not appear to compromise overall cardiac function in this short-term model. None of the identified studies directly report PR interval prolongation or first-degree atrioventricular (AV) block as an adverse effect of SSZ in humans or animal models [35,42,43].

A central finding of our study is the robust attenuation of OS markers by SSZ in both heart and aortic tissues. DOX administration markedly increased MDA in the aorta and elevated the OSI in the myocardium, consistent with its well-documented role in ROS overproduction [7,9]. Concurrently, key antioxidant defenses—including SOD, GSH, and CAT—were significantly depleted in the DOX group, particularly in vascular tissue. SSZ may activate the nuclear factor erythroid 2–related factor 2 (Nrf2) signaling pathway, potentially increasing the expression of antioxidant enzymes such as heme oxygenase-1 (HO-1) and endothelial nitric oxide synthase (eNOS). This mechanism may enhance antioxidant defenses and reduce oxidative damage in vascular tissues. Previous studies have suggested that these effects may involve the extracellular signal-regulated kinase (ERK) and c-Jun N-terminal kinase (JNK) pathways, which are implicated in Nrf2 activation and subsequent antioxidant gene expression [19]. SSZ pretreatment effectively reversed these alterations: aortic MDA levels were normalized, SOD activity was preserved in both heart and aorta, and GSH levels in the aorta were partially restored. These effects are mechanistically plausible given SSZ’s dual capacity to directly scavenge ROS/RNS and modulate redox-sensitive signaling pathways [14]. Although SSZ is known to inhibit the system xc^−^ cystine/glutamate antiporter, potentially limiting GSH synthesis under certain conditions, our data suggest that in the context of acute DOX insult, its net effect is antioxidant [12,13]. This observation may be associated with NF-κB suppression, which has been suggested to reduce NADPH oxidase-driven ROS generation and the downstream inflammatory amplification of oxidative injury [10,11]. In model of ulcerative colitis, SSZ improved antioxidant defenses and reduced inflammation, effects that have been partly attributed to activation of the Nrf2 pathway and suppression of NF-κB signaling [44]. Preclinical studies outside the chemotherapy context further support the biological plausibility of SSZ. In cardiac transplantation models, SSZ reduced ischemia–reperfusion injury and prolonged allograft survival, effects that have been associated with suppression of NF-κB-driven adhesion molecule expression and leukocyte infiltration [43]. Similarly, SSZ has shown protection in renal ischemia–reperfusion models, supporting its tissue-protective potential in acute oxidative–inflammatory insults [45].

Notably, TAS in heart tissue was significantly higher in the SSZ + DOX group than in the DOX group, reinforcing the idea that SSZ bolsters endogenous antioxidant capacity during anthracycline stress. Conversely, TOS and OSI remained elevated in aortic tissue even with SSZ, indicating that vascular compartments may be more vulnerable to persistent oxidative imbalance—a finding that underscores the need for tissue-specific cardioprotective strategies.

Histological evaluation provided compelling morphological validation of SSZ’s protective effects. The DOX group exhibited hallmark features of acute myocardial injury: interstitial edema, hemorrhage, and widespread cardiomyocyte degeneration with eosinophilic cytoplasmic changes and pyknotic nuclei. These findings are consistent with human and experimental models of DOX-induced myocardial injury [4,6]. In stark contrast, the SSZ + DOX group showed near-complete preservation of myocardial architecture, with histopathological scores comparable to controls. This dramatic reduction in structural damage strongly supports the functional and biochemical improvements observed. One study in a rat cardiac transplant model found that SSZ reduced reperfusion injury, as measured by decreased cardiac edema, neutrophil infiltration, and contraction band necrosis. This was associated with reduced expression of inflammatory adhesion molecules and prolonged graft survival, suggesting a protective effect in this specific context [43].

Studies on rodents have shown that doxorubicin-induced structural damage to the aorta can occur in the chronic phase [46]. In our study, DOX induced significant thinning of the aortic tunica intima-media within 24 h, whereas SSZ pretreatment not only prevented this thinning but restored intima-media thickness to levels approaching those of the control group. This vasculoprotective effect may be associated with the reported ability of SSZ to enhance endothelial cell migration and proliferation in placental and vascular tissue models and to suppress adhesion molecule expression through inhibition of NF-κB signaling [18,43]. Another study in high-glucose-exposed rat vessels showed that SSZ reduced endothelial dysfunction and vascular damage by activating antioxidant pathways but did not directly assess cardiomyocyte degeneration, interstitial edema, or hemorrhage in the myocardium [19].

The cardiovascular effects of SSZ remain controversial. While preclinical studies in transplantation and diabetes models demonstrate clear benefits, a randomized trial in patients with established coronary artery disease found no improvement in endothelial function, systemic inflammation, or vascular health markers, and even reported poor tolerability [19,43,47]. In diabetic rat models, it improved endothelial function and reduced oxidative stress in blood vessels [19]. In a mouse model of angiotensin II-induced cardiac remodeling, SSZ actually worsened cardiac dysfunction, hypertrophy, and fibrosis. This negative effect was associated with activation of the Akt signaling pathway, independent of its anti-inflammatory properties [48]. These discrepancies highlight the context-dependent nature of SSZ’s actions: its efficacy appears greatest in acute, inflammation-driven injury but may be neutral or detrimental in chronic pressure-overload or metabolic stress settings. In a randomized controlled trial with patients who had established coronary artery disease, SSZ did not improve endothelial function, BP, or vascular dilation. The drug was also poorly tolerated due to gastrointestinal side effects. There was no significant effect on systemic inflammation or lipid profiles, and no improvement in vascular health markers was observed in most subgroups [47]. In rat heart transplant models, SSZ reduced reperfusion injury and prolonged graft survival, likely by inhibiting inflammatory pathways [43].

In our study, the acute, ROS- and NF-κB-driven nature of DOX-induced myocardial injury may provide a favorable context for the potential cardioprotective effects of SSZ. Based on previous studies, SSZ may inhibit IKK and suppress NF-κB nuclear translocation, thereby potentially reducing the expression of cytokines, chemokines, and adhesion molecules involved in inflammatory cell recruitment and tissue injury. Similar mechanisms have been proposed to contribute to cardioprotection in experimental cardiac allograft models [10,11,43]. Additionally, SSZ’s direct ROS-scavenging capacity and potential activation of the Nrf2 antioxidant pathway may synergistically enhance cellular resilience [14,44].

Several limitations must be acknowledged. First, our model uses a single high-dose DOX regimen, which mimics acute inflammatory myocardial injury but not the cumulative cardiotoxicity seen in clinical oncology. Future studies should evaluate SSZ in chronic, low-dose DOX protocols that better reflect human treatment schedules. Second, we did not assess long-term survival, cardiac function or tumor response—critical factors for clinical translation. Third, the molecular mechanisms underlying SSZ’s effects (e.g., NF-κB phosphorylation, Nrf2 nuclear translocation, ferroptosis modulation) were inferred but not directly measured. Fourth limitation of this study is the absence of an SSZ-only treatment group. Although the primary objective was to evaluate the protective effects of SSZ against DOX-induced cardiotoxicity, inclusion of an SSZ-only group would have allowed assessment of the independent effects of SSZ on cardiovascular, biochemical, and histopathological parameters under physiological conditions. Future studies incorporating this group would further clarify whether the observed effects are attributable solely to protection against DOX-induced injury or also reflect the intrinsic pharmacological actions of SSZ. Fifth, our study focused solely on the acute phase of DOX-induced cardiotoxicity; therefore, long-term outcome data and longitudinal survival rates were not evaluated. Sixth, the assessment was limited to biochemical and histopathological changes; no functional cardiac data, such as echocardiographic parameters or ventricular pressure measurements, were measured. Seventh, our study was conducted exclusively on male rats, which limits the generalizability of our findings to both sexes. Since it is known in the literature that female rodents are more resistant to DOX-induced cardiotoxicity due to the protective effects of estrogen, future studies including female subjects are needed to evaluate potential sex-related differences in the therapeutic efficacy of SSZ. Finally, the optimal dosing, timing, and safety profile of SSZ in combination with anthracyclines remain undefined.

## 5. Conclusions

In summary, our findings provide valuable preliminary evidence that SSZ attenuates acute biochemical and histopathological alterations against DOX-induced acute myocardial injury in rats. These benefits are mediated primarily through the attenuation of OS, preservation of antioxidant defenses, and mitigation of histopathological injury in both myocardial and aortic tissues. While the use of a single high-dose DOX protocol rather than a cumulative chronic regimen, along with the lack of functional cardiac assessments, limits the immediate translational relevance of these findings, the overall data support SSZ as a promising candidate for further evaluation in mitigating acute chemotherapy-induced toxicities. Future investigations are strictly required to focus on chronic cumulative dosing models, long-term functional cardiac outcomes, and combinatorial approaches with established cardioprotectants to fully determine the clinical potential of this repurposed anti-inflammatory agent.

## Figures and Tables

**Table 1:** Rat body and heart weights.

Parameters *	Control Group (*n* = 8)	DOX Group (*n* = 10)	SSZ + DOX Group (*n* = 10)	*p* Value
**Rat experiment starting weight (g)**	386 (294–432)	381 (335–430)	403 (344–467)	0.293
**Rat experiment ending weight (g)**	382 (287–435)	369 (310–402)	388 (320–434)	0.402
**Heart weight (g)**	1.4 (0.9–1.6)	1.4 (1.2–1.5)	1.3 (1.1–1.5)	0.432

* Variables are summarized as medians (minimum–maximum).

**Table 2:** ECG changes.

Parameters (Count)	Control Group (*n* = 8)	DOX Group (*n* = 10)	SSZ + DOX Group (*n* = 10)	*p* *
**Arrhythmia**	0	1	3	0.298
**ST segment depression**	0	1	0	0.999
**ST segment elevation**	0	3	1	0.298
**T wave negativity**	1	1	0	0.715
**Aberrant conduction**	0	0	1	0.999

* Fisher’s exact test.

**Table 3:** Heart histopathological evaluation results.

Groups	Cardiomyocyte Degeneration	Interstitial Edema	Hemorrhage
Control Group	0.0 (0.0–1.0)	0.0 (0.0–2.0)	0.0 (0.0–2.0)
DOX Group	1.0 (0.0–3.0) ^b^	1.0 (0.0–3.0) ^b^	0.0 (0.0–3.0) ^b^
SSZ + DOX Group	0.0 (0.0–1.0) ^a^	0.0 (0.0–1.0) ^a^	0.0 (0.0–2.0) ^a^

Data are expressed as median (minimum–maximum). ^a^ = *p* < 0.05 between the DOX group and the SSZ + DOX group, ^b^ = *p* < 0.05 between the DOX group and the control group.

**Table 4:** Histological score results for tunica intima-media thickness.

Groups	Tunica Intima-Media Thickness (µm)
Control Group	98.8 ± 13.9
DOX Group	93.6 ± 12.2 ^b^
SSZ + DOX Group	101.9 ± 12.6 ^a^

Data are expressed as arithmetic mean ± standard error. ^a^ = *p* < 0.05 between DOX group and SSZ + DOX group, ^b^ = *p* < 0.05 between DOX group and the control group.

## Data Availability

The data presented in this study are available on request from the corresponding author.
